# The Doctor

**Published:** 1904-01-02

**Authors:** 


### "THE DOCTOR."

WE have been asked to state that a number of pirated copies of the above well-known picture by Luke Fildes, R.A., have been introduced from abroad, and are now being circulated in this country. The genuine copyright engraving of this picture is published by Messrs. Thos. Agnew and Sons, of 39B Old Bond Street.

